# Determinants of Vitamin D Status: An Analysis in a Primary Care Setting in Lithuania of Age, Gender and Seasonality

**DOI:** 10.3390/medicina62061172

**Published:** 2026-06-17

**Authors:** Beata Martinkienė, Benedikt Bachmetjev, Rima Piličiauskienė, Gintarė Sragauskienė

**Affiliations:** 1 Faculty of Medicine, Vilnius University, M.K. Ciurlionio 21, LT-03101 Vilnius, Lithuania; 2 Karoliniskiu Clinic, L. Asanaviciutes St. 27a, LT-04318 Vilnius, Lithuania

**Keywords:** vitamin D, vitamin D deficiency, seasons, age factors, sex factors, Lithuania, cross-sectional studies, public health surveillance

## Abstract

*Background and Objectives*: Vitamin D deficiency is a pervasive public health issue in high-latitude regions, yet large-scale population data for the Baltic states remain sparse. This study aimed to determine the prevalence of vitamin D status and identify its primary determinants within a primary care setting in Lithuania. *Materials and Methods*: We conducted a retrospective cross-sectional analysis of serum 25-hydroxyvitamin D [25(OH)D] concentrations from 14,330 unique patients (aged 1–101 years) collected during 2025 at a major clinic in Vilnius. Vitamin D status was categorized according to the Central and Eastern European Expert Consensus thresholds. *Results*: The overall median 25(OH)D concentration was 68.3 nmol/L, placing it in the “insufficiency” range (50–75 nmol/L). Seasonality emerged as the most significant predictor of deficiency; multivariable logistic regression showed a maximal risk reduction in September (OR 0.33; 95% CI: 0.27–0.41) and August (OR 0.34) compared to January, while June and November provided no significant protection. Age-specific analysis revealed a non-linear “U-shaped” distribution: children aged 0–6 years had the highest levels (median ~87–91 nmol/L), likely due to rickets prophylaxis, whereas adolescents (12–18 years) exhibited the highest vulnerability, with approximately 80% suffering from deficiency or insufficiency. Males faced a 13.9% higher likelihood of deficiency than females (OR 1.14; *p* = 0.0036), potentially due to lower rates of elective supplementation. *Conclusions*: These findings suggest that current supplementation strategies successfully protect infants but fail to sustain adequacy through adolescence and adulthood, particularly during the “vitamin D winter.” Targeted public health interventions for adolescents and year-round monitoring are recommended to mitigate the high prevalence of suboptimal vitamin D status in Lithuania.

## 1. Introduction

Vitamin D deficiency represents a major global public health concern, affecting an estimated one billion individuals worldwide across all age groups, geographic regions, and socioeconomic backgrounds [1,2]. Notably, this burden is not limited to regions with low sun exposure. Systematic reviews have demonstrated that deficiency remains prevalent even in countries with year-round sunlight [2]. Vitamin D is primarily linked to calcium and phosphorus metabolism and bone health. Moreover, the clinical significance of vitamin D extends beyond skeletal health. Studies have demonstrated associations between low vitamin D status and increased risk of several chronic conditions, including certain cancers, type 2 diabetes, cardiovascular disease, and autoimmune disorders [3,4,5]. There remains debate in the recent literature regarding which 25(OH)D concentrations should define deficiency and sufficiency. According to the latest Endocrine Society guidelines, published in 2024, healthy adults younger than 75 years require no routine screening for 25(OH)D in the general population and empiric vitamin D supplementation [6]. In contrast, the Institute of Medicine considers the minimal 25(OH)D concentration of 50 nmol/L as physiologically adequate for at least 97.5% of the population [7]. Although prior regional data heavily rely on these historical reference limits [8,9], our study adopted the Central and Eastern European consensus thresholds. These guidelines were specifically developed for populations sharing Lithuania’s geographic, climatic, and nutritional characteristics [9,10].

Within Europe, the prevalence of vitamin D deficiency remains high and exhibits marked geographical heterogeneity [11,12]. Paradoxically, Northern European countries such as Finland, Sweden, and Norway often report higher population 25(OH)D concentrations compared to Central and Eastern European countries, where vitamin D deficiency is considerably more prevalent [12,13,14]. Lithuania, situated at approximately 54–56° N latitude, falls into a zone where cutaneous synthesis of vitamin D via ultraviolet B (UVB) radiation is physiologically limited from October through March, restricting endogenous production to a narrow seasonal window [11]. Combined with limited dietary vitamin D sources, low habitual consumption of oily fish, and the absence of a mandatory vitamin D food fortification policy, endogenous and exogenous vitamin D supply in Lithuania remains insufficient for most of the year [15,16]. Furthermore, lifestyle factors common across European populations at similar latitudes, including predominantly indoor occupational activities, reduced time spent outdoors during the cold season, and limited deliberate sun exposure even in summer, may further contribute to the risk of vitamin D deficiency in the Lithuanian population [17].

Despite the recognized public health significance of vitamin D deficiency, population-level data on 25(OH)D concentrations in Lithuania remain limited. The largest population-based study to date was conducted by Bleizgys and Kurovskij in 2018, including 9581 subjects, and demonstrated a high overall prevalence of vitamin D deficiency (67%). In contrast, a high prevalence of vitamin D hypervitaminosis was observed among young children [10]. The largest study investigating a pediatric population was performed by Butkute et al., who analyzed 2008 samples from the Children’s Hospital, and reported that 51.3% of the study population were vitamin D deficient [18]. Other existing studies have focused on specific subgroups. Strazdiene et al. examined elderly individuals and found that 72.2% of women and 65.6% of men were vitamin D deficient [19]. Smaller Lithuanian studies targeting specific subgroups—such as military conscripts [20], female school graduates [21], young males [22], and patients with rheumatoid arthritis [23]—similarly report a high baseline prevalence of suboptimal vitamin D levels.

We aimed to, firstly, determine the prevalence of suboptimal vitamin D status within a primary care cohort; secondly, evaluate the independent and combined effects of age, sex, and season on serum 25(OH)D concentrations using multivariable regression; and thirdly, compare our findings with previously published Lithuanian data over the past decade.

## 2. Materials and Methods

### 2.1. Study Design and Setting

We conducted a retrospective cross-sectional study analyzing serum 25-hydroxyvitamin D [25(OH)D] concentrations in a primary care setting in Lithuania. Anonymized data were retrieved from the laboratory information system of Karoliniskiu Clinic in Vilnius, Lithuania. To ensure the representation of the baseline population health status, we analyzed historical laboratory data collected over a period from 2 January 2025 to 21 December 2025. The study adhered to the principles of the Declaration of Helsinki and followed the Strengthening the Reporting of Observational Studies in Epidemiology (STROBE) guidelines.

The serum concentration of total 25-hydroxyvitamin D was determined using a two-step competitive binding immunoenzymatic assay (paramagnetic particle, chemiluminescent immunoassay). All the analyses were performed on the UniCel DxI Immunoassay System (Beckman Coulter, Inc., Brea, CA, USA), utilizing sheep monoclonal anti-25(OH) vitamin D antibodies and a vitamin D analog–alkaline phosphatase conjugate to achieve high analytical sensitivity and specificity.

### 2.2. Study Participants and Sampling

The initial dataset contained 15,421 laboratory entries. To avoid selection bias introduced by repeated measurements (e.g., follow-up tests after prescribed supplementation), we applied a strict data deduplication protocol. Unique patients were identified using a combination of birth date, gender, and the referring physician ID. Only the first chronologically recorded test (baseline measurement) for each unique individual was included in the final analysis. Subsequent tests for the same patient were excluded to reflect the natural prevalence of vitamin D status before intervention.

The final study sample consisted of 14,330 unique subjects, ranging in age from infancy to centenarians. No exclusions were made based on comorbidities or clinical department.

### 2.3. Variables and Measurements

In the absence of specific national guidelines for vitamin D reference ranges in Lithuania, we adopted the classification criteria proposed by the Central and Eastern European Expert Consensus Statement (2022). This consensus, developed by a panel of experts from the region (including Poland, Latvia, and Estonia), aligns with major international epidemiological studies [9]. Specifically, we utilized the consensus-endorsed threshold of <30 nmol/L for deficiency and >75 nmol/L for sufficiency to ensure our findings are comparable. Based on this clinical data, vitamin D status was classified into the following categories:

- Severe Deficiency: <30 nmol/L.
- Deficiency: 30–50 nmol/L.
- Insufficiency: 50–75 nmol/L.
- Sufficiency (Optimal): 75–125 nmol/L.
- Hypervitaminosis: 125–374 nmol/L.
- Toxicity Risk: >374 nmol/L.

### 2.4. Data Analysis

Statistical analysis and data visualization were performed using the R Statistical Software (v4.x, R Foundation for Statistical Computing) within the RStudio (V. 2024.12.1+563) environment. The tidyverse and ggplot2 packages were utilized for data manipulation and graphical representation.

Descriptive Statistics: Normality of the data distribution was assessed visually using histograms, kernel density estimation (KDE) plots, and Q-Q plots, as well as statistically using the Shapiro–Wilk test. Since the distribution of 25(OH)D levels showed a positive skew (right-tailed), continuous variables are reported strictly as medians with interquartile ranges (IQR). Categorical variables are presented as absolute numbers (*n*) and percentages (%).

Comparative Analysis: To compare the 25(OH)D levels between two independent groups (e.g., males vs. females), the non-parametric Mann–Whitney U test was applied. For comparisons across multiple age groups, the Kruskal–Wallis H test was used, followed by pairwise post hoc analysis. The Chi-square (χ^2^) test was employed to compare the frequency of clinical deficiency categories between groups.

Regression Analysis: To identify predictors of vitamin D deficiency (<50 nmol/L), we performed binary logistic regression. A multivariate logistic regression model was fitted to calculate adjusted Odds Ratios (aORs) with 95% Confidence Intervals (CIs), estimating the independent risk associated with male gender and specific age groups.

A *p*-value of <0.05 was considered statistically significant for all the tests.

### 2.5. Ethical Considerations

This study utilized fully anonymized, retrospective laboratory data retrieved from the clinic’s database. In accordance with the Law on Ethics of Biomedical Research of the Republic of Lithuania and GDPR Recital 26, projects that process exclusively and irreversibly anonymized retrospective data—where all direct and indirect personal identifiers are removed and individual identification is permanently impossible—do not fall under the legal definition of biomedical research on human subjects. Because no human subjects were directly involved, exposed to interventions, or identifiable, mandatory Institutional Review Board (IRB) or Lithuanian Bioethics Committee approval was not legally required for this retrospective analysis. The study was conducted in strict adherence to the Ethical Principles of Non-Biomedical Research Involving Human Health, ensuring absolute data protection and identity de-identification.

## 3. Results

### 3.1. Descriptive Statistics

A total of 14,330 unique subjects were retained in the final analysis following the exclusion of duplicates. The study population spanned a broad demographic spectrum (Table 1), ranging from 1 to 101 years of age. The median age of the study population was 37.0 years (IQR: 30.0; Mean: 38.8 years). Formal normality testing and kernel density estimation (KDE) demonstrated that the overall age distribution was non-Gaussian. While the female cohort displayed a broader, more unimodal distribution, the male cohort exhibited a distinct bimodal pattern confirmed by KDE, with a primary peak in early childhood (ages 0–6 years) and a secondary peak in young adulthood (ages 20–35 years).

**Figure 1:**
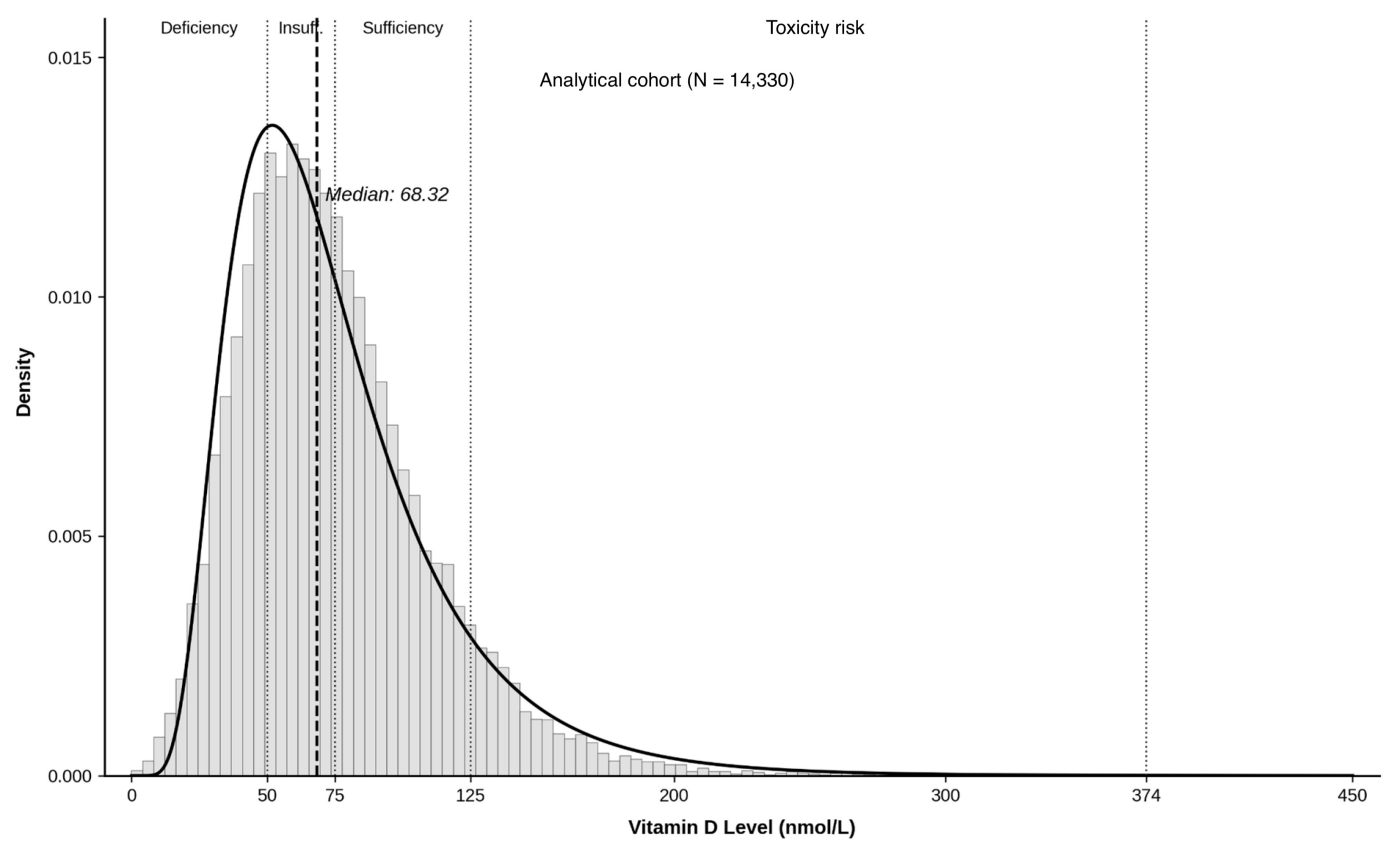
Distribution of serum 25(OH)D levels in the study population.

The analysis of serum 25(OH)D concentrations confirmed a non-normal distribution (Figure 1), showing a significant right-tailed positive skew (Kolmogorov–Smirnov test statistic D = 0.12, *p* < 0.001; Shapiro–Wilk test statistic W = 0.89, *p* < 0.001).

### 3.2. 25(OH)D Distribution by Age

Regarding gender composition (Table 1), the study population was predominantly female (72.3%, *n* = 10,359), while males accounted for 27.7% (*n* = 3971) of the participants.

**Figure 2:**
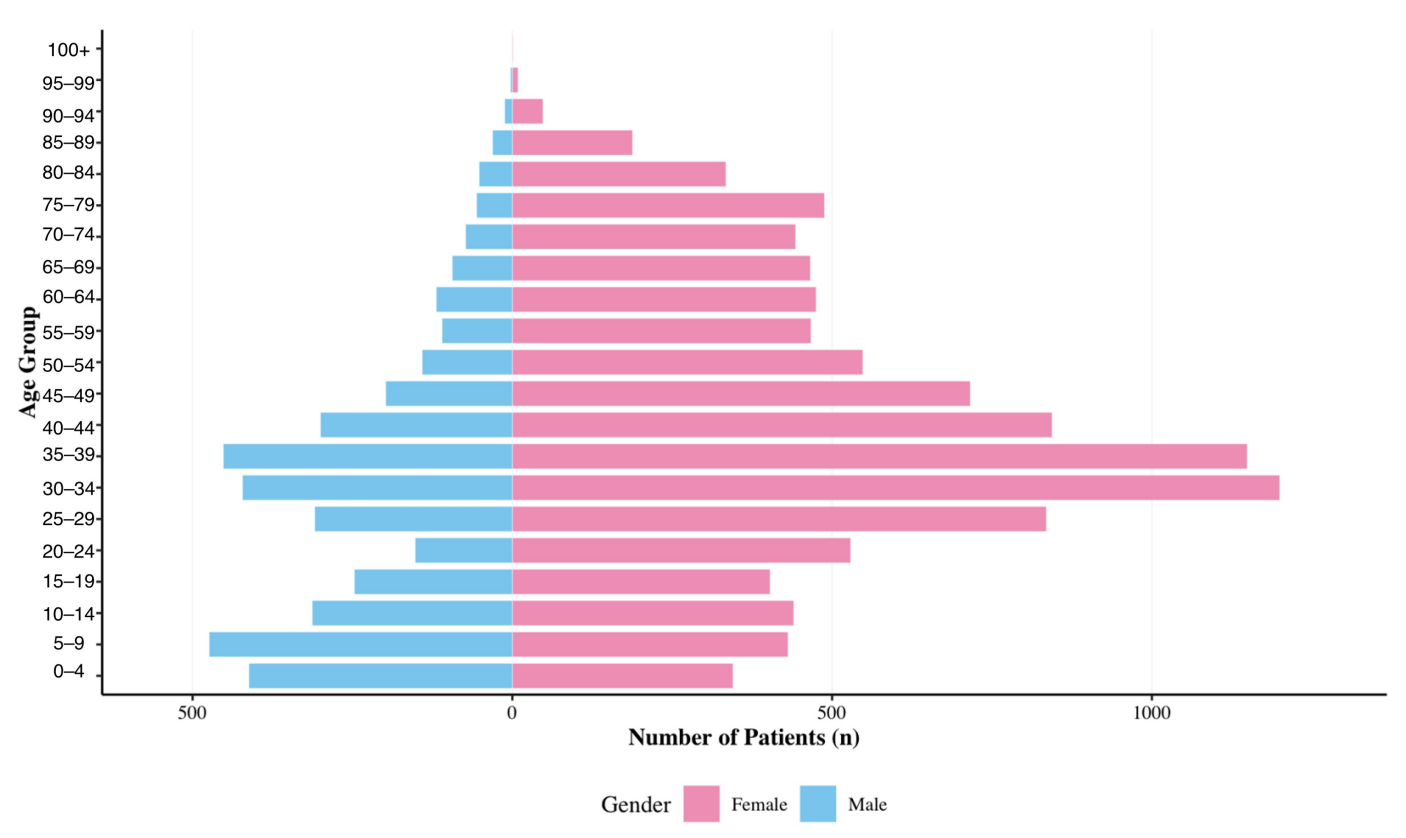
Population pyramid illustrating the demographic structure of the study cohort by age and gender.

The age distribution differed significantly by gender (Table 1), with females generally being older. The median age for females was 39.1 years (IQR: 28.0–57.9), compared to 31.0 years (IQR: 11.3–43.0) for males. The female cohort spanned from 1.0 to 102.0 years, showing a broader distribution in older age brackets. In contrast, the male cohort (range: 1.0 to 97.2 years) was more heavily concentrated in younger demographics (Figure 2).

Data collection was distributed relatively evenly (Table 2) throughout the year, with peak activity observed in May (9.8%) and April (9.6%), while the lowest volume of tests was recorded in December (6.4%).

**Figure 3:**
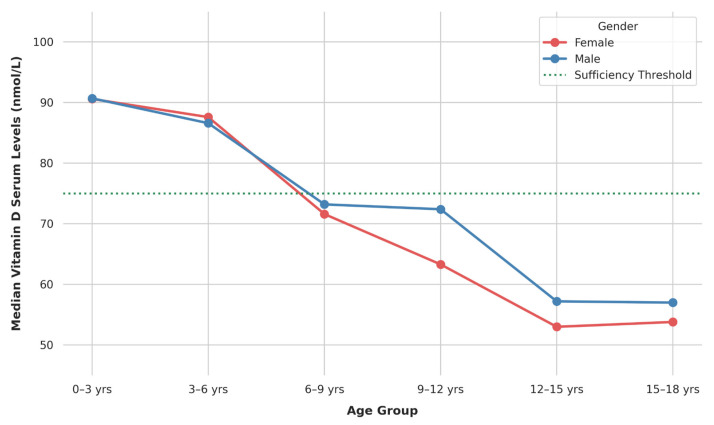
25(OH)D serum levels (nmol/L) across pediatric age groups and gender.

The biggest changes in the 25(OH)D levels were recorded in patients under the age of 18. This is why a more precise analysis of this group was conducted. The pediatric analysis (Table 3) demonstrated a strong inverse relationship between age and the 25(OH)D serum levels. The highest concentrations were recorded in the 0–3 years group, where both females (median: 90.6 nmol/L) and males (median: 90.7 nmol/L) exhibited the highest baseline status. Conversely, the lowest concentrations were found in the 12–15 and 15–18 years cohorts, with the female median levels dropping to 53.0 nmol/L and 53.8 nmol/L, respectively. While the 25(OH)D levels remained comparable between genders in early childhood, a divergence became apparent in the 9–12 years group, where males maintained a higher median (72.4 nmol/L) compared to females (63.3 nmol/L; *p* < 0.01). Overall, the median values showed a consistent decline from over 90 nmol/L in toddlers to approximately 53–57 nmol/L in adolescents (Figure 3).

**Figure 4:**
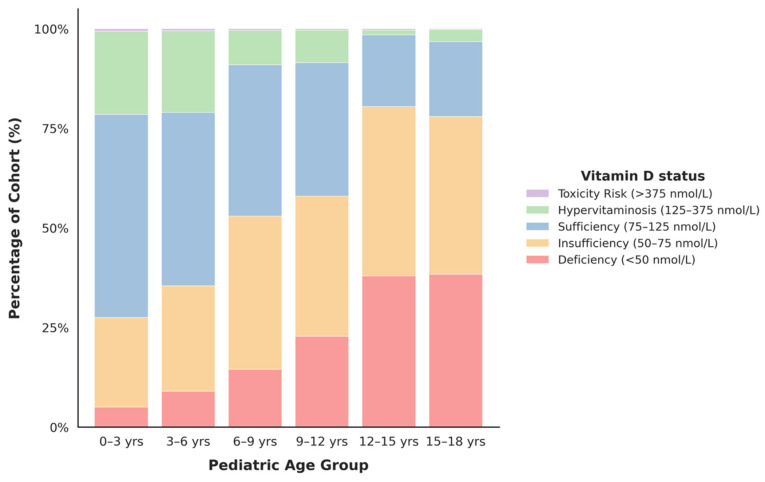
Distribution of vitamin D status from deficiency to hypervitaminosis across pediatric age groups.

The categorical analysis (Figure 4) reveals a significant shift in vitamin D status as children age, transitioning from a high prevalence of sufficiency and hypervitaminosis in early childhood to widespread deficiency in adolescence. In the 0–3 years group, 51.0% of the children achieved sufficiency, while a notable 20.9% showed hypervitaminosis. Similarly, the 3–6 years group maintained high levels, with 20.5% showing hypervitaminosis. However, as age increased, the proportion of children with adequate levels decreased sharply. By the 12–15 and 15–18 years age groups, deficiency (less than 50 nmol/L) and insufficiency (50–75 nmol/L) became the dominant clinical states, together affecting approximately 80% of those cohorts. Specifically, deficiency reached its peak in the 15–18 years group at 38.4%, while the hypervitaminosis group fell to just 3.0% in the same age bracket. Cases with a risk of toxicity (>375 nmol/L) remained extremely rare across all the pediatric groups, occurring in 0.5% or less of the study population.

### 3.3. 25(OH)D Distribution by Gender

The analysis of vitamin D status revealed a high level of consistency between genders, although the female participants exhibited slightly higher central values (Table 3). The median 25(OH)D concentration for females was 68.9 nmol/L and 66.9 nmol/L for males. Both medians fall within the “insufficiency” range (50–75 nmol/L) as defined by the Central and Eastern European Expert Consensus.

The median values for females (68.9 nmol/L) and males (66.9 nmol/L) are numerically close. Males exhibited a wider interquartile range (IQR = 37.8 nmol/L) compared to females (IQR = 34.0 nmol/L), suggesting a broader spread of results and a higher frequency of extreme values within the male cohort.

In both groups, the mean is significantly higher than the median (a difference of 6.0 nmol/L for females and 7.2 nmol/L for males), confirming a right-skewed distribution driven by outliers with high 25(OH)D concentrations.

### 3.4. Vitamin D Distribution by Seasonality and Age

**Figure 5:**
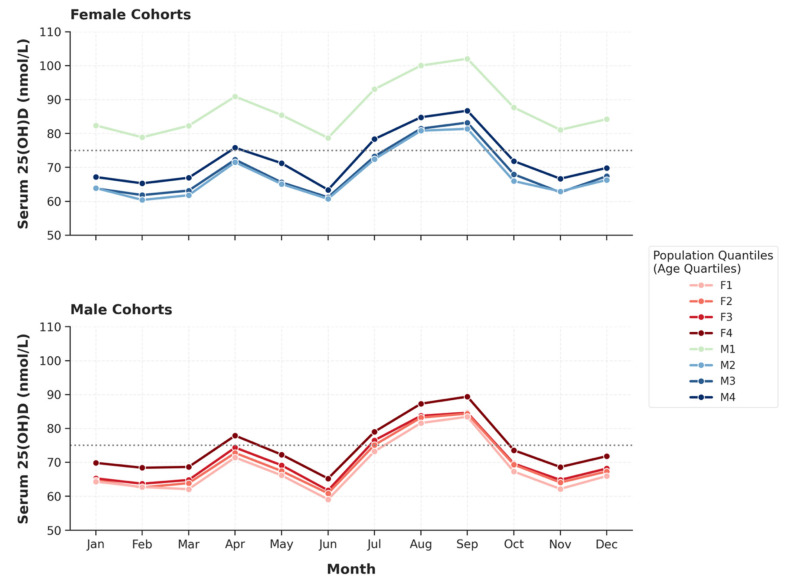
Comparative analysis of median vitamin D distribution by gender and quantile. Values represent monthly medians, where F and M represent females and males, respectively, and 1–4 represent age quartiles.

The study population of 14,330 participants was stratified into eight distinct cohorts (Figure 5) based on gender and age quartiles (F1–F4 and M1–M4). Age divisions were established using the population’s interquartile range: Q1 included individuals up to 24 years, Q2 and Q3 spanned from 24 to 54 years, and Q4 represented those above 54 years.

Seasonal vitamin D dynamics follow a distinct biphasic pattern (Figure 5). The primary peak occurs across all groups during August and September, reaching maximum median concentrations in the M1 (90.1 nmol/L) and F4 (87.4 nmol/L) cohorts. A secondary, less pronounced peak is observed in mid-spring (April), followed by a transient decline in May and June.

Troughs are most evident during the winter months and the start of summer. Middle-age groups (M2 and M3) show the most significant fluctuations, with medians dropping to 60.0–60.4 nmol/L in June. While older cohorts and youngest males (F4 and M1) maintain higher baselines, they follow the same trend: an April rise, a June dip, and a definitive annual peak in late summer.

**Figure 6:**
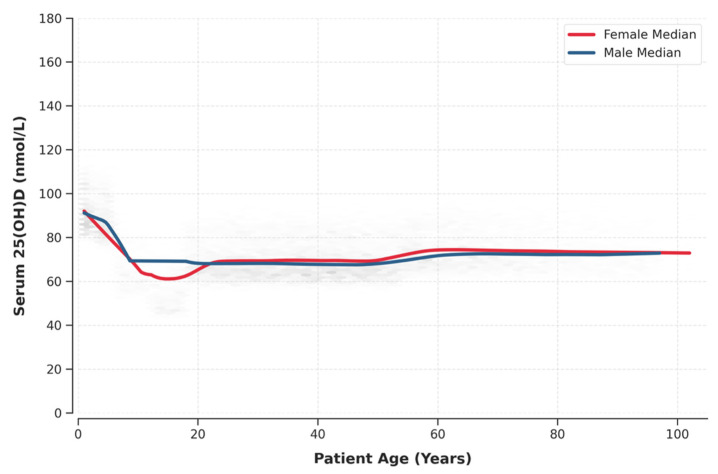
Serum 25(OH)D median values across population.

Conversely, the lowest median concentrations were recorded in the young adult cohort (Q2), which presented a median of 65.9 nmol/L (*p* < 0.001). These results indicate a non-linear distribution where serum 25(OH)-vit D levels are highest in the youngest segment (reaching a median of 90.6 nmol/L) and the oldest population segments (stabilizing at a median 73.1 nmol/L), while reaching their nadir in the adolescent and young adult groups, dropping to a median of approximately 60.1 nmol/L for females and 74.1 nmol/L for males (Figure 6).

### 3.5. Multivariable Predictors and Odds Ratios of Vitamin D Deficiency

To validate the primary analysis, a multivariable logistic regression model was developed. The model (Table 4) identified age, gender, and seasonality as significant predictors of vitamin D deficiency. Gender emerged as a notable risk factor, with males exhibiting a 13.9% higher likelihood (OR 1.140; *p* = 0.0036) of falling into the deficiency range compared to females. Age showed a statistically significant but negligible protective effect (OR 0.997; *p* < 0.001).

Seasonality exerted the most substantial impact on risk levels. September and August provided the highest protective effect (Table 4), reducing risk by 67% and 65.9% respectively. A secondary period of significant risk reduction occurred in July (−46%) and April (−38.3%). In contrast, months such as June and November showed no statistically significant change in risk. The data confirms that while biological factors like gender are influential, the magnitude of risk is predominantly driven by seasonal variation.

## 4. Discussion

### 4.1. Vitamin D Status Across Age Groups

To our knowledge, our study represents the largest population-based assessment of vitamin D status conducted in Lithuania. In this study, the overall median serum 25(OH)D concentration was 68.3 nmol/L, placing the average within the “insufficiency” range (50–75 nmol/L) and confirming a widespread suboptimal vitamin D status in Lithuania. The 25(OH)D concentrations varied substantially across age groups, with the highest median levels observed in the youngest and oldest groups, and the lowest in adolescents aged 15–18 years. Age is a well-established determinant of vitamin D status, and the European Calcified Tissue Society has identified young children, adolescents, and older adults as key risk groups [14]. In a pooled analysis of standardized data from over 55,000 Europeans spanning all age groups, Cashman et al. reported an overall prevalence of vitamin D deficiency (<30 nmol/L) of 13%, with considerable variation by age group and latitude [12]. However, direct comparison of age-stratified data across studies is complicated by substantial heterogeneity in age group classification. For instance, in Lithuanian studies Bleizgys and Kurovskij employed broad decade-wide groupings, Butkute et al. divided children only into those under and over 2 years, while European studies used varying pediatric categories, for example, 0–1, 2–5, 6–10, 11–15, 16–20, 21–30, 31–40, etc., years in the Czech cohort, 0–1, 1–6, 7–12, 13–18 years in the Belgian cohort [10,18,24,25]. Similarly, definitions for adult and elderly cohorts ranged from >50 to >70 years across different studies [10,26,27,28,29]. Despite these methodological differences, the literature consistently points to two vulnerable age periods: young children, who tend to have the highest concentrations largely driven by supplementation practices, and adolescents, who show the steepest rise in deficiency prevalence. Among older adults, the findings are more heterogeneous, with some studies reporting increasing concentrations with age in supplement-using populations [26], while others document high deficiency rates in the institutionalized elderly [19,26,29]. We examined these patterns below in two subgroups: children and older adults, where the clinical implications are most pronounced.

#### 4.1.1. Vitamin D Status in Pediatric Age Groups

Our study revealed that the highest 25(OH)D median concentrations occurred in the 0–3 years age group, where both females (90.6 nmol/L) and males (90.7 nmol/L) exhibited the highest status. A similar pattern was observed in the 3–6 years age group, with median concentrations of 87.6 nmol/L for females and 86.6 nmol/L for males. Our findings echo those of Bleizgys and Kurovskij, who noted that young children under nine had the highest 25(OH)D levels [10], and Butkute et al., who explored optimal thresholds in Lithuanian pediatric cohorts [18]. While young children face a higher probability of hypervitaminosis, absolute hypervitaminosis remains extremely rare [10,18]. This localized trend toward higher baseline levels in early childhood is linked to established national policies for routine infant rickets prophylaxis [30,31], although individual adherence to these guidelines could not be verified in our data.

Data from other European cohorts (Ukraine, the Czech Republic, Belgium, and Romania) consistently mirror this pattern: infants and toddlers up to age six are at the lowest risk for clinical deficiency [24,25,32,33], with hypervitaminosis occurring only in isolated, exceptional cases [25,32,33,34].

Conversely, pediatric vitamin D deficiency rates increase with age, demonstrating a prominent vulnerability during adolescence [10,25,32,33,34]. This progressive decline across regional and international cohorts is associated with the cessation of routine supplementation after infancy, paired with a general reduction in parental lifestyle oversight [10,25,33,34]. Beyond supplementation, pan-European multi-country data confirm that time spent outdoors, dietary choices, and ambient UVB radiation remain critical modifiable determinants of baseline pediatric vitamin D adequacy [17].

#### 4.1.2. Vitamin D Status in Older Adults

In our cohort, the oldest age group exhibited the highest baseline 25(OH)D levels. This contrasts with other Lithuanian data, such as the study by Strazdiene et al., where the lowest levels and severe deficiency were concentrated among elderly individuals aged 80 and older [19]. It also deviates from the modest trend toward declining concentrations with advancing age reported by Bleizgys and Kurovskij [10]. International data present a highly varied picture due to varying cutoff values across cohorts. While some findings, like the Dutch study by Verbakel et al., align with our observation of increasing vitamin D status in older demographics [26], cohorts in Great Britain, Ireland, and Austria document widespread insufficiency and deficiency in older and institutionalized populations [27,28,29]. Multivariable risk factor evaluations across European cohorts confirm that this demographic variance is deeply multifactorial. Key negative determinants of 25(OH)D levels in older adults include seasonal winter depletion, institutionalization, advanced old age, higher BMI, physical inactivity, and smoking [26,28,35]. Conversely, higher continuous levels are driven by targeted over-the-counter supplement use, fish oil consumption, frequent oily fish intake, and higher cumulative UVB radiation exposure [26,28,35].

It should be noted that the apparent trend toward higher continuous 25(OH)D levels in the oldest cohort (Q4) is partially driven by the right-skewed nature of the data and the presence of high-dose supplementation outliers within that specific demographic, whereas the overall risk of clinical deficiency tracking below the 50 nmol/L threshold remains high as demonstrated by the regression model.

### 4.2. Sex Differences in Vitamin D Status

In our cohort, females exhibited a modestly higher median 25(OH)D concentration, with male sex associated with a 13.9% higher likelihood of clinical deficiency (OR 1.140; *p* = 0.0036). This contrasts with a previous Lithuanian population-based cohort where men demonstrated significantly higher baseline concentrations than women [10].

Our findings also show divergent patterns when compared to broader European literature. While some national surveys in Germany, Finland, and Ukraine observed no statistically significant sex differences among adult populations [32,36,37], cohorts in the Netherlands documented higher mean 25(OH)D concentrations among males [26]. Conversely, and consistent with our regression model, a high-volume Czech study reported a significant negative association between male sex and circulating 25(OH)D concentrations [24], while Ukrainian adolescent data similarly identified higher deficiency rates in young males [32].

This gender-based divergence between continuous trends and binary regression risk highlights how right-skewed data can mask baseline vulnerabilities. The elevated levels in young male pediatric cohorts pull the continuous data trends upward, whereas the multivariable regression model evaluates the probability of dropping below the clinical threshold of 50 nmol/L, where adult males exhibit a higher risk.

Several factors explain this discrepancy. First, differential supplementation behavior is influential: women are more engaged in preventive health and exhibit significantly higher odds of regular vitamin D use compared to males [34]. Second, the marked sex imbalance in our sample (72.3% female vs. 27.7% male) may introduce selection and indication bias, as women are more likely to undergo routine biochemical testing. Third, residual confounding from sex-specific differences in BMI, adiposity distribution, lifestyle habits, and occupational sun exposure further influence circulating 25(OH)D concentrations.

Furthermore, the marked demographic asymmetry in our sample, characterized by a higher proportion of older females compared to older males, could potentially confound a crude comparison of baseline 25(OH)D levels between genders. Because older individuals in this setting frequently exhibit higher continuous concentrations due to targeted supplementation, this age distribution imbalance could artificially skew unadjusted gender metrics. However, our reliance on a multivariable logistic regression model mitigates this confounding effect by adjusting for age and gender simultaneously, thereby confirming that male gender remains an independent risk factor for clinical deficiency.

Hormonal factors also contribute, as exogenous estrogen exposure from oral contraceptives has been shown to modulate vitamin D metabolism and elevate median 25(OH)D concentrations [37]. Collectively, these elements indicate that the observed sex differences are highly multifactorial and context-dependent rather than purely biological.

### 4.3. Seasonal Variation

Lithuania is situated within the high-latitude “vitamin D winter” zone, where UVB radiation is insufficient [11]. Pan-European pooled data confirm a clear seasonal divide in vitamin D status, with deficiency rates doubling in winter compared to summer [12]. Prior Lithuanian data are highly consistent, tracking an annual nadir in January–April and a peak in August–September [10]. In local pediatric cohorts, this seasonal dependency emerges rapidly once routine infant supplementation ceases after age two, presenting as a significant spring drop [18].

Our results are broadly consistent with these findings, tracking a primary peak in late summer across all demographics and a secondary hypervitaminosis in April, followed by a transient May–June decline. This mirrors the post-winter transitional dip seen in older Lithuanian children before sustained summer UVB synthesis fully commences [18].

Specific meteorological anomalies during the 2025 study period may have further modulated these seasonal dynamics. Localized data from the Lithuanian Hydrometeorological Service indicate that April was the sunniest month of the year, while winter and summer recorded fewer sunshine hours than normal [38]. This unusually sunny spring favored earlier cutaneous synthesis across high-latitude boundaries [11], whereas below-average summer sunshine explains the unexpected June dip, particularly in middle-aged working cohorts. The complete lack of statistical risk reduction in June and November further highlights the limits of the narrow regional UVB window [11].

This profound seasonal instability is consistently demonstrated across broader European literature. Longitudinal, cohort, and regional studies in the Netherlands, Slovenia, Central Europe, the Czech Republic, and Belgium confirm severe late-winter depletions and a high reliance on seasonal sun exposure or targeted supplementation to prevent structural drop-offs [12,24,25,26,39,40].

## 5. Conclusions

In this retrospective cross-sectional study of 14,330 individuals—the largest clinic-based assessment of 25(OH)D status in Lithuania to date—the overall median serum 25(OH)D concentration was 68.3 nmol/L, falling within the insufficiency range by Central and Eastern European consensus criteria.

Seasonality was the strongest determinant of deficiency risk, with August–September associated with up to 67% reduced odds compared to January, while June and November showed no significant protective effect—underscoring that even nominally warm months do not reliably support adequate vitamin D synthesis at Lithuanian latitudes. Male sex was independently associated with modestly higher odds of deficiency (OR 1.14; *p* = 0.004), potentially reflecting differences in supplementation behavior rather than biology alone. Age effects were non-linear: children aged 0–6 years exhibited the highest concentrations, likely attributable to infant supplementation policies, while adolescents aged 12–18 years had the poorest status, with deficiency and insufficiency affecting approximately 80% of this group.

These findings highlight adolescents as a priority population for targeted supplementation interventions beyond early childhood and support the rationale for routine October–March supplementation strategies within this and similar ambulatory cohorts in Vilnius. As the data originate from a single primary care center, these patterns specifically reflect the studied clinic population and should not be assumed to represent nationwide metrics. Future prospective, multi-center, nationally representative studies incorporating data on supplementation use, BMI, dietary intake, and sun exposure are warranted to further clarify the contributions of these modifiable factors across the broader Lithuanian population.

### Limitations of the Study

Several limitations of this study should be noted. First, the retrospective cross-sectional design means we can only show associations, not prove cause and effect. Second, our data did not include information on lifestyle factors that influence vitamin D, such as diet, Body Mass Index (BMI), skin type, or the specific amount of time spent in the sun. Third, because we used laboratory records, we could not track whether the participants were taking over-the-counter vitamin D supplements before their blood test. While we removed duplicate tests to focus on baseline levels, some patients might have already started supplementing, which could skew the results. Fourth, since the data comes from a single clinic in Vilnius, the findings may not represent the entire country or people living in rural areas. Finally, because the study covers only one year, we cannot account for weather changes between different years that might affect sun exposure.

Finally, while our large sample size provided high statistical power, the available database lacked the clinical covariates required for complex non-linear spline modeling or advanced interaction testing, meaning that residual confounding between age, sex, and seasonal synthesis cannot be entirely ruled out.

## Figures and Tables

**Table 1:** Demographic characteristics and serum 25(OH)D concentrations of the study population.

	Total Cohort (*n* = 14,330)	Female Cohort (*n* = 10,359)	Male Cohort (*n* = 3971)
Sample Size, *n* (%)	14,330 (100.0%)	10,359 (72.3%)	3971 (27.7%)
Age (Years)			
Median (IQR)	37.0 (30.0)	39.1 (29.9)	31.0 (31.7)
95% CI	38.4–39.1	42.1–42.9	28.4–29.8
Range (Min–Max)	1.0–101.0	1.0–102.0	1.0–97.2
Serum 25(OH)D (nmol/L)			
Median (IQR)	68.3 (37.0)	68.9 (34.0)	66.9 (37.8)
95% CI	74.0–75.2	74.2–75.5	72.9–75.3
Range (Min–Max)	2.3–500.7	2.3–500.7	2.5–498.2

**Table 2:** Monthly distribution of vitamin D testing.

Month	Jan	Feb	Mar	Apr	May	Jun	Jul	Aug	Sep	Oct	Nov	Dec	Total
Count (n)	1212	1174	1225	1373	1399	1143	1231	1085	1188	1255	1131	914	**14,330**
Percentage (%)	8.5%	8.2%	8.5%	9.6%	9.8%	8.0%	8.6%	7.6%	8.3%	8.8%	7.9%	6.4%	**100.0%**

**Table 3:** Serum 25(OH)D concentrations stratified by gender.

Gender	*n*	Median	SD	95% CI
Female	10,359	68.9	34.0	74.2–75.5
Male	3970	66.9	37.8	72.9–75.3

**Table 4:** Multivariate logistic regression analysis of factors associated with vitamin D deficiency.

Factor	Odds Ratio (OR)	95% CI	Risk Change (%)	*p*-Value
Age (per year)	0.997	0.995–0.998	−0.3%	<0.001
Male	1.140	1.040–1.240	+13.9%	0.0036
January	1.00	Reference	0.0%	-
February	0.752	0.625–0.905	−24.8%	0.0025
March	0.772	0.643–0.925	−22.8%	0.0052
April	0.617	0.514–0.740	−38.3%	<0.001
May	0.730	0.612–0.870	−27.0%	<0.001
June	0.954	0.798–1.140	−4.6%	0.6013
July	0.540	0.447–0.652	−46.0%	<0.001
August	0.341	0.275–0.422	−65.9%	<0.001
September	0.330	0.267–0.405	−67.0%	<0.001
October	0.707	0.592–0.844	−29.3%	<0.001
November	0.925	0.777–1.100	−7.5%	0.3832
December	0.821	0.681–0.989	−17.9%	0.0387

## Data Availability

The dataset supporting the conclusions of this publication is proprietary and will not be publicly shared. However, additional information regarding the study methodology and analysis can be provided upon reasonable request to the corresponding author.
